# Will artificial intelligence solve the human resource crisis in healthcare?

**DOI:** 10.1186/s12913-018-3359-4

**Published:** 2018-07-13

**Authors:** Bertalan Meskó, Gergely Hetényi, Zsuzsanna Győrffy

**Affiliations:** 1 Institute of Behavioural Sciences, Semmelweis University, Nagyvárad square 4, Budapest, H-1089 Hungary; 2 Semmelweis University, Nagyvárad square 4, Budapest, H-1089 Hungary; 3 The Medical Futurist Institute, Budapest, Hungary

**Keywords:** Digital health, Artificial intelligence, Human resources, Healthcare, Medicine, Physicians

## Abstract

Artificial intelligence (AI) has the potential to ease the human resources crisis in healthcare by facilitating diagnostics, decision-making, big data analytics and administration, among others. For this we must first tackle the technological, ethical and legal obstacles.

The human resource crisis is widening worldwide, and it is obvious that it is not possible to provide care without workforce. How can disruptive technologies in healthcare help solve the variety of human resource problems? Will technology empower physicians or replace them? How can the medical curriculum, including post-graduate education prepare professionals for the meaningful use of technology? These questions have been growing for decades, and the promise of disruptive technologies filling them is imminent with digital health becoming widespread. Authors of this essay argue that AI might not only fill the human resources gap, but also raises ethical questions we need to deal with today.

While there are even more questions to address, our stand is that AI is not meant to replace caregivers, but those who use AI will probably replace those who don’t. And it is possible to prepare for that.

## Background

### There is a human resources crisis in healthcare today

Every second post-millennial believes that they will work together with robots and artificial intelligence (AI) within 10 years [1]. We examine the questions of what this desire means for the future of the workforce, and whether it has any implications for the healthcare industry.

The healthcare workforce crisis is due to at least three major issues: doctor shortages worldwide, the aging and burnout of physicians and a higher demand for chronic care. An effective system *depends on the availability, accessibility, acceptability and quality of its health workers* [2]*.* It is estimated that the needs-based shortage of healthcare workers globally is about 17.4 million, and the aging workforce is an additional challenge [3].

With the increase of life expectancy (the population over 65 years is expected to double by 2030) and the number of chronic illnesses, the demand towards the healthcare system is also constantly growing. Consequently, the lack of access to care and the differing quality are general worldwide. 400 million people lack access to one or more essential health services, and five billion people do not have access to safe, affordable surgical and anaesthesia care when needed [4].

One in three physicians are over 55 years of age, and a third of physicians are expected to retire in the next decade. As the new generation of medical professionals looks for a limited number of working hours, a speciality without having to be on-call and an acceptable work-life balance, this wish for a more controllable lifestyle might further increase shortages.

Because of the growing number of chronic patients and physicians being overloaded due to shortages, burnout is increasing [5]. It can be the cause of somatic symptoms, substance use, psychological and sleep disorders; as well as maladaptive coping strategies. As physicians’ wellbeing is linked to the quality and the safety of outcomes, this further fuels challenges.

The human resource crisis is widening across the globe, and it is obvious that without a capable workforce there is no way to provide quality care. How can disruptive technologies in healthcare help solve the variety of human resource problems? Will technology empower physicians or replace them? How can the medical curriculum, including post-graduate education prepare professionals for the meaningful use of technology?

## Main text

### Artificial intelligence shows potential for filling these gaps

These gaps have been growing for decades, and the promise of technology filling them is imminent with digital health becoming widespread. Authors of this essay argue that AI might not only fill the human resource gaps, but also raise ethical questions we need to assess today.

As Nick Bostrom describes in his book *Superintelligence*, AI is divided broadly into three stages: artificial narrow intelligence (ANI), artificial general intelligence (AGI) and artificial superintelligence (ASI) [6]. In the next decade, ANI has the highest chance of being used in the medical practice for analyzing large datasets, finding new correlations and generally supporting caregivers’ jobs.

An obvious first step is clearing up the definitions around AI to stop its misuse in medical communication. Here we attempt at providing short definitions for the most common expressions.

#### Artificial narrow intelligence

It is good at performing a single task, such as playing chess, poker or Go, making purchase suggestions, online searches, sales predictions and weather forecasts.

#### Artificial general intelligence

It can understand and reason its environment similarly as a human being would do, therefore it’s also known as human-level AI.

#### Artificial superintelligence

According to Nick Bostrom, it’s smarter than the best humans in every field from scientific creativity to general wisdom and social skills.

#### Supercomputers

A supercomputer is a computer with a high level of computing performance used for resource-intensive tasks, such as machine and deep learning.

#### Machine learning

Machine learning is one of the many subsets of AI that refers to creating programs based on data as opposed to programming rules. A software that learns from large sets of relevant data (e.g. feeding it with a lot of radiology images and letting it discover recurring patterns).

#### Deep learning

It is a specialized subset of machine learning that uses neural networks, an artificial replication of the structure and functionality of the brain. It’s efficient at various tasks such as image recognition, natural language processing and translation. The performance of deep learning algorithms continues to improve as datasets grow significantly which means the bigger the dataset, the better the outcome and efficiency improves.

Various companies and organizations have already demonstrated how AI can contribute to improve the quality of care and/or decreasing costs [7].

Deepmind Health launched a cooperation with the Moorfields Eye Hospital NHS Foundation Trust to improve eye treatment by mining one million anonymized eye scans with the related medical records. IBM launched Watson Oncology to provide clinicians with evidence-based treatment options and an advanced ability to analyze the meaning and context of structured and unstructured data in clinical notes and reports.

In the Netherlands, Zorgprisma Publiek helps caregivers and hospitals avoid unnecessary hospitalizations of patients by analyzing the digital invoices obtained from insurance companies with IBM Watson in the cloud.

In radiology, the Medical Sieve project aims at building the next-generation “cognitive assistant” with analytical, reasoning capabilities and a range of clinical knowledge. Such an assistant would be able to analyze radiology images to detect medical issues. In genomics, Deep Genomics helps identify linkages to diseases in large data sets of genetic information and medical records.

In pharmaceutical research, Atomwise uses supercomputers to find new therapies speeding up clinical trials that take sometimes more than a decade and cost billions of dollars. As an example, Atomwise found two drugs predicted by the company’s AI technology which may significantly reduce Ebola infectivity in less than a day of research, instead of years.

Deep learning algorithms have demonstrated to be able to help the diagnosis of conditions in cardiology, dermatology and oncology [8, 9].

Arterys already received FDA clearance for its AI-assisted cardiac imaging system in 2017. AI supported messaging apps and voice controlled chatbots can also help take off the burden on medical professionals regarding easily diagnosable health concerns or quickly solvable health management issues. Safedrugbot is a chat messaging service that offers assistant-like support to health professionals who need appropriate information about the use of drugs during breastfeeding.

AI-based services could facilitate more accurate diagnoses, administration, decision-making, big data analytics, post-graduate education, among others. However, we need to emphasize that practicing medicine is not a linear process. Every single element and parameter cannot be translated into a programming language. Moreover, there is no clinical trial or peer-reviewed data about the data points that contribute to a medical decision. It’s clear that AI is not the ultimate solution for all the challenges healthcare faces today. Although, in many areas, its use is inevitable and advantageous in supporting caregivers’ job.

However, a tight framework from regulatory agencies would further stop companies from providing false hope for patients claiming more than what they can deliver and prove. Moreover, the FDA has assembled a team of computer scientists and engineers to help oversee and anticipate future developments in AI-driven medical software [10]. These are encouraging steps forward, but the range of ethical, legal and social implications of using AI in healthcare are even beyond the scope of what we can deal with today.

## Debate

### A myriad of questions we need answers for

Resource-poor regions will face challenges while adopting AI. On one hand, the cost of disruptive technologies might be too high for underdeveloped countries, pushing them further behind in improving healthcare. This still stands if we consider that the use of new technologies could be cost-effective in the long run. If a country invests into buying an AI-based decision-support system, it could help physicians make better decisions, thus leading to fewer number of unnecessary hospitalizations, which reduces costs.

On the other hand, underdeveloped countries can be more open to policy changes that would facilitate the adoption of such technologies, which could lead to a more widespread adoption than in developed regions. Examples include how Rwanda opened up its emergency care system to Zipline that produces and operates medical drones across the country.

Regarding caregivers in general, do we need narrow or general AI to provide better care? What elements of their repetitive tasks, such as note taking or administrative duties could AI ease, and which ones such as diagnosis, treatment or monitoring could it facilitate?

Technology could also offer solutions to improve the access to care [11]. With AI, it is easier for medical professionals to care for a larger number of patients. AI tools help them make better diagnostic decisions, improve treatment outcomes and reduce medical errors. AI could also take part in solving HR issues, such as recruiting and selecting the potential healthcare workforce. It’s important to point out that the HR crisis cannot be solved by only developing technologies for physicians. All healthcare professionals must be involved.

However, AI does not cover the whole process of treatment: empathy, proper communication and the human touch are still equally essential. No application, software or device can replace personal connection and trust. The role of the human physician is inevitable, but AI could be a very useful cognitive assistant.

AI also means a paradigm shift in the doctor-patient relationship. As digital health transforms the well-known doctor-patient hierarchy into an equal level partnership, what happens with the autonomy that has been the essence of care [12]? Who is responsible if an AI-assisted medical decision causes harm to a patient? Most doctors use online tools to help with research. Is there really a difference when it comes to using AI? Should AI be handled as another tool, such as a stethoscope or as an individual entity?

On the patients’ side, will they stick to the human touch when shortages simply do not give them a chance to meet a physician in person for every medical issue? What if AI algorithms can mimic empathy either through an app or a chatbot? It’s not yet known whether they will accept the use of AI in decision-making and learn its use in their care.

On the level of society, will it help shift focus from treatment to prevention? Will AI increase the cost of care? Will doctors and medical professionals be more efficient, because AI handles some of the time-consuming tasks? Will doctors provide better care in underdeveloped regions with the use of AI? And generally, how will it (if at all) change the current structures of insurance policies?

## Conclusions

If we might be brave enough to articulate a vision, the authors of this debate article think that AI will eventually be evidence-based, widespread and affordable. Physicians have been translating the data they measured with rudimentary tools, like stethoscopes or blood pressure cuffs, and they will keep on doing the same with digital tattoo-like sensors and AI. We think this technology will reduce costs in providing care, making it faster and more efficient leading to a change in the medical profession that will involve more tasks related to creativity and critical thinking than time-consuming repetitions.

In about 20 years, 50% of jobs will be outdated or not needed anymore, and healthcare is not an exception [13]. While AI demonstrates significant potentials in improving diagnostics [14–16], it will probably not solve the HR crisis in healthcare, or at least it will not start with that. The chance for improving the job environment and conditions of physicians is higher, which can eventually lead to a general improvement in the quality of care. If it becomes able to take over important tasks from medical professionals, it might even bring forward a renaissance era in the doctor-patient relationship.

While there are even more questions to address, our stand is that AI is not meant to replace medical professionals, but the ones using AI will probably replace those who don’t. We also think that it is every caregivers’ duty to prepare for a future like that.

## Abbreviations

AGI: Artificial general intelligence
AI: Artificial intelligence
ANI: Artificial narrow intelligence
ASI: Artificial superintelligence
FDA: Food and Drug Administration
